# Chondroitin Sulfate Ameliorates Hypertension in Male Offspring Rat Born to Mothers Fed an Adenine Diet

**DOI:** 10.3390/antiox13080944

**Published:** 2024-08-02

**Authors:** You-Lin Tain, Chih-Yao Hou, Guo-Ping Chang-Chien, Shu-Fen Lin, Chien-Ning Hsu

**Affiliations:** 1 Division of Pediatric Nephrology, Kaohsiung Chang Gung Memorial Hospital, Kaohsiung 833, Taiwan; tainyl@cgmh.org.tw; 2 College of Medicine, Chang Gung University, Taoyuan 330, Taiwan; 3 Institute for Translational Research in Biomedicine, Kaohsiung Chang Gung Memorial Hospital, Kaohsiung 833, Taiwan; 4 Department of Seafood Science, National Kaohsiung University of Science and Technology, Kaohsiung 811, Taiwan; chihyaohou@webmail.nkmu.edu.tw; 5 Super Micro Mass Research and Technology Center, Cheng Shiu University, Kaohsiung 833, Taiwan; guoping@csu.edu.tw (G.-P.C.-C.); linsufan2003@csu.edu.tw (S.-F.L.); 6 Institute of Environmental Toxin and Emerging-Contaminant, Cheng Shiu University, Kaohsiung 833, Taiwan; 7 Center for Environmental Toxin and Emerging-Contaminant Research, Cheng Shiu University, Kaohsiung 833, Taiwan; 8 School of Pharmacy, Kaohsiung Medical University, Kaohsiung 807, Taiwan; 9 Department of Pharmacy, Kaohsiung Chang Gung Memorial Hospital, Kaohsiung 833, Taiwan

**Keywords:** hydrogen sulfide, hypertension, chondroitin sulfate, gut microbiota, chronic kidney disease, developmental origins of health and disease (DOHaD)

## Abstract

Pregnant women with chronic kidney disease (CKD) face increased risks of adverse outcomes in their adult offspring. Offspring rats born to dams fed an adenine diet develop hypertension, coinciding with dysregulated hydrogen sulfide (H_2_S) and nitric oxide (NO) pathways, as well as alterations in gut microbiota. Chondroitin sulfate (CS) is a multifunctional food known for its diverse bioactivities. As a sulfate prebiotic, CS has shown therapeutic potential in various diseases. Here, we investigated the protective effects of maternal CS supplementation against hypertension in offspring induced by an adenine diet. Mother rats were administered regular chow, 0.5% adenine, 3% CS, or a combination throughout gestation and lactation. Maternal CS supplementation effectively protected offspring from hypertension induced by the adenine diet. These beneficial effects of CS were connected with increased renal mRNA and protein levels of 3-mercaptopyruvate sulfurtransferase, an enzyme involved in H_2_S production. Furthermore, maternal CS treatment significantly enhanced alpha diversity and altered beta diversity of gut microbiota in adult offspring. Specifically, perinatal CS treatment promoted the abundance of beneficial microbes such as *Roseburia hominis* and *Ruminococcus gauvreauii*. In conclusion, perinatal CS treatment mitigates offspring hypertension associated with maternal adenine diet, suggesting that early administration of sulfate prebiotics may hold preventive potential. These findings warrant further translational research to explore their clinical implications.

## 1. Introduction

Chronic kidney disease (CKD) impacts around 10% of the global population, including pregnant women [1,2]. Maternal CKD not only leads to pregnancy complications but also increases the risk of adverse outcomes in adult offspring [1,2]. Adverse conditions during pregnancy can affect the likelihood of developing adult-onset diseases, a concept now known as the developmental origins of health and disease (DOHaD) or developmental programming [3,4]. This phenomenon proposes a potential to prevent adult diseases by intervening in developmental processes before they clinically manifest, termed reprogramming [5].

The adenine diet model of CKD in rodents is a well-established model used to study human CKD [6]. In prior work, we observed that rats born from dams fed an adenine diet developed hypertension and kidney hypertrophy by 12 weeks of age, which are early signs of CKD [7,8]. Our findings also suggest that maternal adenine diet-induced hypertension in offspring may be linked to deficiencies in nitric oxide (NO), impaired hydrogen sulfide (H_2_S) signaling, as well as alterations in gut microbiota and their metabolites [7,8].

Chondroitin sulfate (CS) is a sulfated glycosaminoglycan frequently found in animal cartilage and connective tissues [9]. CS has demonstrated anti-inflammatory, antioxidant, anti-obesity, anti-cancer, and prebiotic properties [9]. Due to its diverse bioactivities, CS has been used to treat osteoarthritis and as a functional food for various other diseases [9,10,11]. Since CS can be utilized by gut microbes [12], its therapeutic effects may be mediated through the regulation of gut microbiota and derived metabolites [13]. Notably, CS supplementation can induce H_2_S production by modulating gut microbiota [14,15].

In addition to bacterial origins, H_2_S can be enzymatically synthesized from L-cysteine by three enzymes: 3-mercaptopyruvate sulfurtransferase (3MST), cystathionine β-synthase (CBS), and cystathionine γ-lyase (CSE) [16]. Previous studies have revealed that exposure to a maternal adenine diet resulted in reduced renal 3MST protein abundance in adult rat offspring [8]. Given H_2_S’s involvement in the pathogenesis of CKD and hypertension, targeting the H_2_S pathway emerges as a potential preventive strategy for hypertension with developmental origins [17]. CS also exhibits anti-inflammatory and antioxidant properties by regulating NO [18], a key player in maternal adenine diet-induced offspring hypertension [7].

Given its multifaceted functions, CS treatment has shown benefits against many diseases in humans and animal models [9,10,11,12,13,14,15]. Based on this background, we examine whether CS supplementation throughout pregnancy and lactation can prevent offspring hypertension induced by a maternal adenine diet. We also explored the protective mechanisms of maternal CS treatment, particularly focusing on H_2_S signaling, NO, and gut microbiota.

## 2. Materials and Methods

### 2.1. Animal Experiments

This study was granted an animal license by the Institutional Animal Ethics Committee at our hospital (Permit # 2020110202) and conformed to the ARRIVE guidelines. For breeding purposes, timed-pregnant Sprague Dawley (SD) rats were obtained from BioLASCO Taiwan Co., Ltd. (Taipei, Taiwan). CKD was induced by feeding the dam a diet containing 0.5% adenine for three weeks before pregnancy, as previously documented [7].

Dams in the model of CKD were divided into four groups (*n* = 3 per group), with different feeding patterns, as follows: the CN group was given an AIN-93G diet (D10012G, RESEARCH DIETS Inc., New Brunswick, NJ, USA); the AD group was given an AIN-93G diet containing 0.5% adenine; the CNCS group was given an AIN-93G diet containing 3% chondroitin sulfate (Han-Sient Trading Co., Ltd., New Taipei City, Taiwan); and the ADCS group was given an AIN-93G diet containing 0.5% adenine plus 3% CS. The dosage and route of CS were selected based on prior rodent studies [14]. After birth, each dam’s litter was standardized to eight pups to ensure uniform pup growth. Given that males are more predisposed to hypertension compared to females [19], the investigation included only male offspring.

Blood pressure (BP) was measured using the Kent Scientific CODA system (Torrington, CT, USA) in offspring from 3 to 12 weeks of age. The rats underwent a 1-week acclimation period to tail-cuff inflation and restraint before the measurements were taken, as previously described [7,8]. At 12 weeks of age, a total of 32 rats (*n* = 8 per group) were killed. Before sacrifice, fresh stool samples were collected and kept in a freezer. Blood samples were collected in heparinized tubes via cardiac puncture. After centrifugation, plasma was separated, divided into Eppendorf tubes, and stored in a −80 °C freezer for subsequent analysis. The kidneys were excised, and the cortex and inner medulla were dissected, immediately snap-frozen, and subsequently stored at −80 °C.

### 2.2. H_2_S-Generating Enzymes

The expression of three H_2_S-generating enzymes, CSE, CBS, and 3MST, in the offspring’s kidneys was determined using quantitative PCR (qPCR) and Western blot. Two-step qPCR was conducted using Quantitect SYBR Green PCR Reagents (Qiagen, Valencia, CA, USA) on a thermal cycler (iCycler, Bio-Rad, Hercules, CA, USA) as we described previously [7,8]. Ribosomal 18S was utilized as a housekeeping gene. For CSE (accession number: NM_017074.2), a forward primer 5′ CGCACAAATTGTCCACAAAC 3′ and a reverse primer 5′ GCTCTGTCCTTCTCAGGCAC 3′ were used. For CBS (accession number: NM_012522.2), a forward primer 5′ ATGCTGCAGAAAGGCTTCAT 3′ and a reverse primer 5′ GTGGAAACCAGTCGGTGTCT 3′ were used. For 3MST (accession number: NM_138843.2), a forward primer 5′ GGCTCAGTAAACATCCCATTC 3′ and a reverse primer 5′ TGTCCTTCACAGGGTCTTCC 3′ were used. All samples were run in duplicate. The comparative threshold cycle (CT) method was employed for the relative quantification of gene expression [20].

Western blotting was performed on kidney samples from offspring. Kidney cortex tissues were homogenized, and equal volumes of protein (200 µg per gel well) were loaded into each well. Following transferring the proteins from the gel to the membrane, Ponceau S staining (PonS, Sigma-Aldrich, St. Louis, MO, USA) was used to normalize protein loading. Membranes were then incubated with primary antibodies: rabbit polyclonal and mouse monoclonal antibodies against CSE (Proteintech Group Inc., Rosemont, IL, USA), CBS (Abnova, New Taipei City, Taiwan), and 3MST (Novus Biologicals, Centennial, CO, USA). Immunostains were quantified using integrated optical density (IOD) analysis with Quantity One Analysis software (Bio-Rad Labs, Hercules, CA, USA). The relative protein abundance was calculated as the ratio of IOD to PonS, correcting for variations in protein loading.

### 2.3. NO Parameters

Measurements of plasma NO-related parameters were made on an Agilent 1100 HPLC system (Agilent Technologies Inc., Santa Clara, CA, USA) with O-phthalaldehyde/3-mercaptopropionic acid as a derivatization agent and fluorescence detection. These NO-related parameters include L-arginine (the substrate for nitric oxide synthase), L-citrulline (the byproduct of NO synthase reaction and the precursor of L-arginine), and symmetric and asymmetric dimethylarginine (SDMA and ADMA, both endogenous inhibitors of nitric oxide synthase). The ratio of L-arginine to ADMA was computed to assess the availability of NO [21].

### 2.4. Plasma Short Chain Fatty Acids (SCFAs)

Since short chain fatty acids (SCFAs) are significant metabolites derived from gut microbiota and are linked to BP, we further investigated their concentrations in the offspring’s plasma [22]. Plasma concentrations of acetic acid, propionic acid, butyric acid, isobutyric acid, isovaleric acid, and valeric acid were determined by GC-MS (QP2010; Shimadzu, Kyoto, Japan) with a flame ionization detector, as detailed in previous studies [7]. A 2 µL aliquot of each sample was injected into the column. The inlet temperature was maintained at 200 °C, while the detector temperature was set to 240 °C. Each analysis had a total run time of 17.5 min.

### 2.5. 16S rRNA Gene Sequencing and Analysis of Gut Microbiota Composition

We utilized 16S rRNA gene sequencing to investigate how CS modulates gut microbiota, leveraging its ability to offer a comprehensive profile of bacterial communities [23]. Its procedure primarily involves sample collection and DNA extraction, PCR amplification, sequencing, and bioinformatics analysis. DNA was extracted from stool samples and sent for 16S rRNA sequencing to Biotools Co., Ltd. in New Taipei City, Taiwan [7]. The full-length 16S genes encompassing V1–V9 hypervariable regions were amplified using barcoded primers, followed by preparation of a multiplexed SMRTbell library (PacBio, Menlo Park, CA, USA) for sequencing. A phylogenetic tree, illustrating the relationship of representative sequence variants (ASVs) sequences, was constructed using QIIME 2′s phylogeny FastTree [24,25]. We calculated two alpha diversity metrics, Faith’s phylogenetic diversity (PD) index and the Shannon index. For beta diversity, the Analysis of Similarities (ANOSIM) and the principal coordinate analysis (PCoA) with unweighted UniFrac distances were employed to compare bacterial composition differences between groups. Linear discriminant analysis effect size (LEfSe) was utilized to detect taxa exhibiting differential abundance [26].

### 2.6. Statistics

Data were shown as the mean ± the standard error of the mean (SEM). Weights, BP, and biochemical parameters were compared using two-way analysis of variance (ANOVA) followed by post hoc comparisons using the Tukey test. A *p*-value < 0.05 was considered statistically significant. Statistical analysis was carried out by SPSS 17.0 (SPSS Inc., Chicago, IL, USA).

In metabolomics analysis for alpha diversity, the Wilcoxon test was used to analyze whether the differences in species diversity between two groups were significant. The ANOSIM used R-value and *p*-value to compare the similarity between groups. An R-value close to 0 represented no significant differences in inter-group and intra-group. An R-value close to 1 showed that inter-group differences were greater than intra-group differences. The *p*-value represented the confidence level of the statistical analysis; *p* < 0.05 reflects a statistically significant difference. Additionally, statistical methods were used to detect species with significant differences in microbial communities between groups, and multiple hypothesis testing and false discovery rate (FDR) analysis were performed to evaluate the observed significant differences. FDR (*q*-value) < 0.05 was considered significant. The LEfSe analysis was examined by the Wilcoxon test.

## 3. Results

### 3.1. Offspring Outcomes

**Figure 1:**
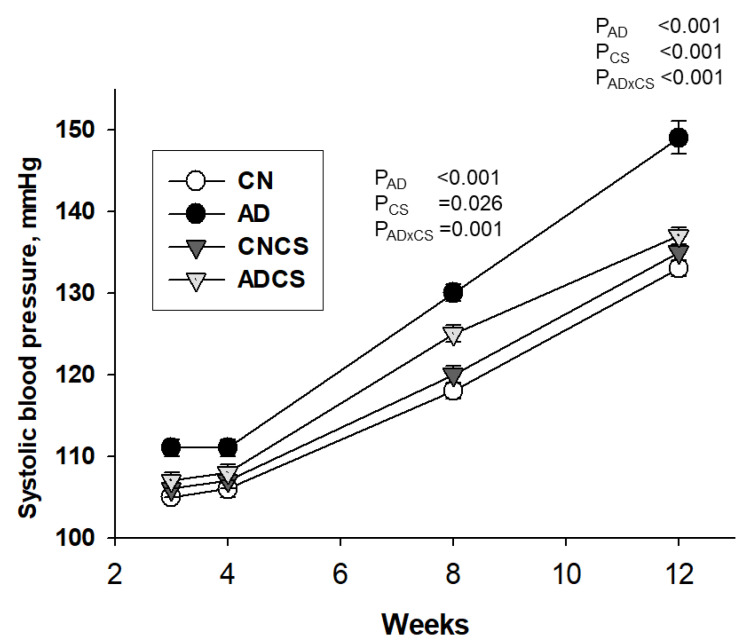
Effects of maternal adenine diet (AD) and chondroitin sulfate (CS) on systolic blood pressure in offspring from Week 3 to 12. N = 8/group; CN = standard diet; AD = standard diet containing 0.5% adenine; CNCS = standard diet containing 3% chondroitin sulfate; ADCS = standard diet containing 0.5% adenine plus 3% CS; AD × CS = interaction of AD × CS.

No puppies died after birth. There was a significant effect of adenine diet (*P*_AD_ = 0.007) or CS treatment (*P*_CS_ = 0.046) on the body weight in offspring (Table 1). Although left kidney weight and combined kidney weight were higher in the AD group compared to the CN group, adenine diet had no effect on the ratios of left and combined kidney-to-body weight. Systolic blood pressure (SBP) in offspring was measured using the tail-cuff method at various ages, as depicted in Figure 1. Maternal adenine diet resulted in increased SBP between 8 to 12 weeks of age (Both *P*_AD_ < 0.001), a condition prevented by CS treatment. Maternal adenine diet increased plasma creatinine concentration in 12-week-old offspring (*P*_AD_ = 0.002), irrespective of whether their dams were treated with CS or not (Table 1). Taken together, these findings indicate that prenatal adenine diet increased SBP and plasma creatinine levels in adult offspring. Maternal CS administration significantly improved hypertension but did not provide protective effects for plasma creatinine levels.

### 3.2. H_2_S Pathway

**Figure 2:**
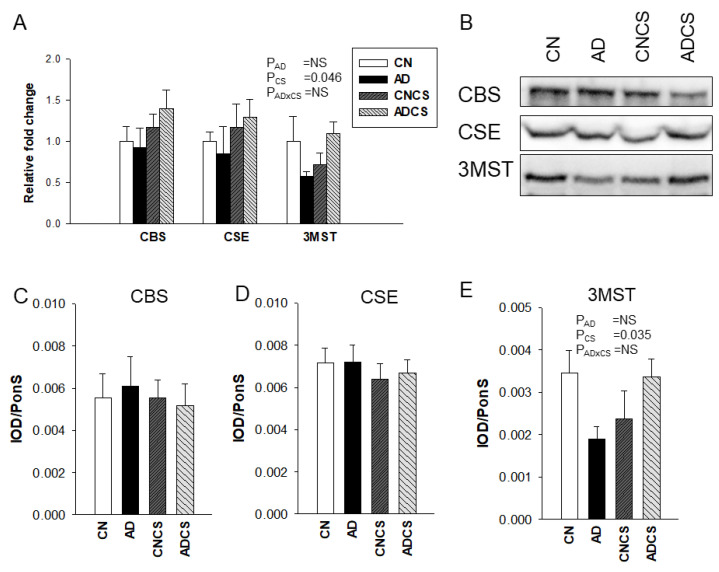
(**A**) Renal mRNA expression of H_2_S-generating enzymes; (**B**) representative Western blot protein bands illustrate immunoreactivity to CBS, CSE, and 3MST. The renal cortical protein abundance of (**C**) CBS (61 kDa), (**D**) CSE (45 kDa), and (**E**) 3MST (52 kDa) was quantified. N = 8/group; CN = standard diet; AD = standard diet containing 0.5% adenine; CNCS = standard diet containing 3% chondroitin sulfate; ADCS = standard diet containing 0.5% adenine plus 3% CS; AD × CS = interaction of AD × CS; NS = not significant.

To determine the influence of maternal adenine diet and chondroitin sulfate administration on the H_2_S pathway, we measured mRNA expression and protein levels of H_2_S-generating enzymes in the offspring’s kidneys. As shown in Figure 2, no differences were detected for renal mRNA expression and protein levels of H_2_S-producing enzymes CBS and CSE between the four groups. Maternal CS treatment had an effect to increase mRNA (*P*_CS_ = 0.046) and protein abundance (*P*_CS_ = 0.035) of 3MST in offspring kidneys.

### 3.3. NO Pathway

As summarized in Table 2, maternal adenine diet caused a decrease in plasma L-citrulline (*P*_AD_ = 0.024) and L-arginine (*P*_AD_ < 0.001). No differences in ADMA and SDMA were observed between the four groups, except there was an interaction between the adenine diet and CS treatment (*P*_AD×CS_ = 0.035). Additionally, maternal adenine diet reduced the L-arginine-to-ADMA ratio (AAR), an index of NO availability in the AD group (*P*_AD_ < 0.001). This reduction was restored by maternal CS treatment (*P*_CS_ = 0.044).

### 3.4. Differences in Microbiota Composition

**Figure 3:**
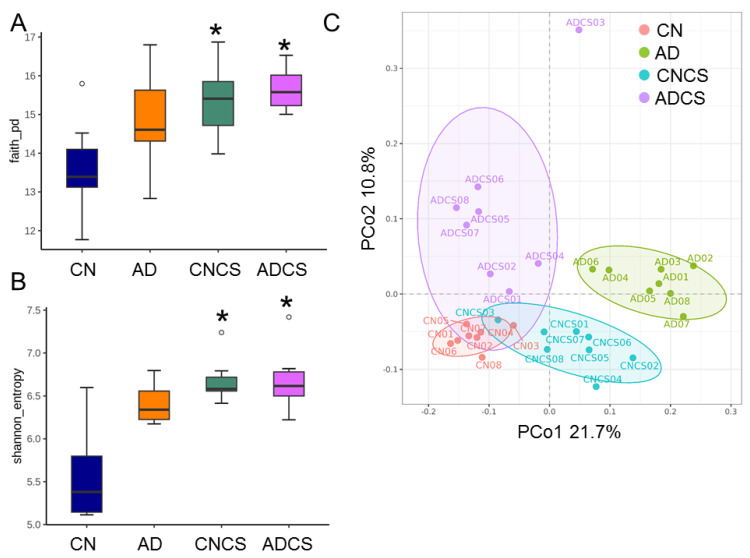
Box plots were generated to illustrate (**A**) Faith’s phylogenetic diversity (PD) index and (**B**) Shannon index, depicting alpha diversity. * *p* < 0.05 by the Wilcoxon test. Outliers are denoted by dots. (**C**) Principal coordinate analysis (PCoA) plots were used to visualize beta diversity, with each data point representing one sample and each color corresponding to a different group. CN = standard diet; AD = standard diet containing 0.5% adenine; CNCS = standard diet containing 3% chondroitin sulfate; ADCS = standard diet containing 0.5% adenine plus 3% CS.

Alpha diversity analysis utilized Faith’s PD index (Figure 3A) and the Shannon index (Figure 3B) to evaluate species richness and evenness. Maternal administration of chondroitin sulfate significantly increased both indices in the CNCS and ADCS groups compared to the CN group, although maternal adenine diet had minimal impact on the AD group.

Beta diversity analysis (Figure 3C), illustrated through PCoA plots, was performed to depict the phylogenetic distance among intestinal bacterial communities. The analysis indicated distinct clustering among the four groups, which was confirmed by ANOSIM demonstrating significant differences between groups (All *p* < 0.05).

**Figure 4:**
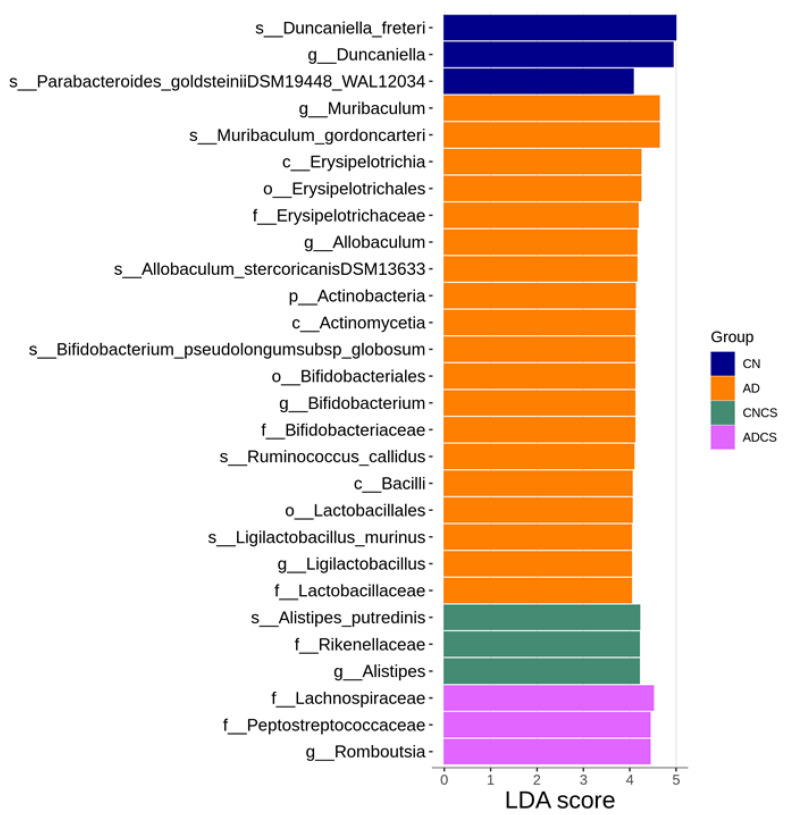
Linear discriminant analysis effect size (LEfSe) was employed to identify taxa that were significantly differentially abundant between groups. Taxa with a linear discriminant analysis (LDA) score greater than 4 were primarily highlighted. The color of the horizontal bar represents the respective group. CN = standard diet; AD = standard diet containing 0.5% adenine; CNCS = standard diet containing 3% chondroitin sulfate; ADCS = standard diet containing 0.5% adenine plus 3% CS.

We then applied LEfSe analysis to identify differentially abundant taxa between groups (Figure 4). The AD group showed a significant increase in the abundance of genus *Muribacculum*, *Allobaculum*, *Bifidobacterium*, and *Ligilactobacillus*. Additionally, LEfSe analysis revealed an increase in the proportion of the genus *Alistipes* in the CNCS group and *Romboutsia* in the ADCS group.

**Figure 5:**
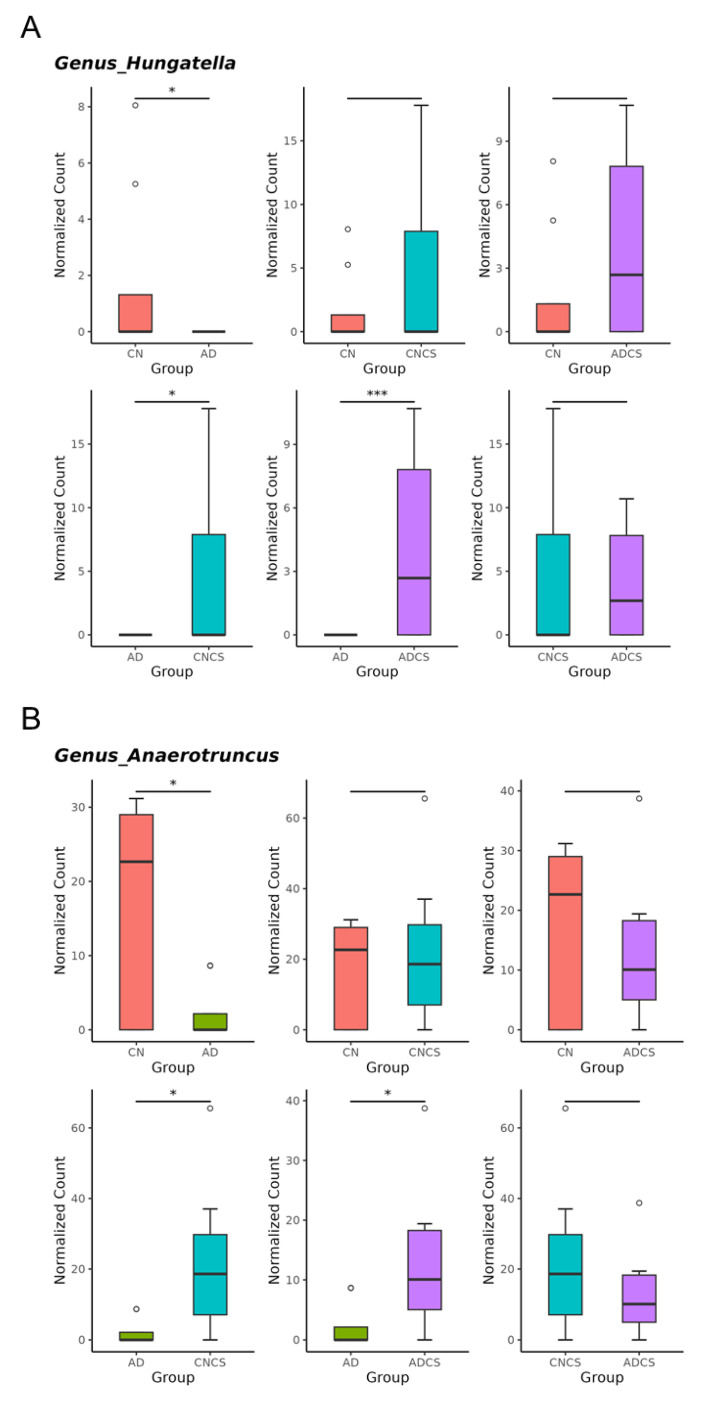

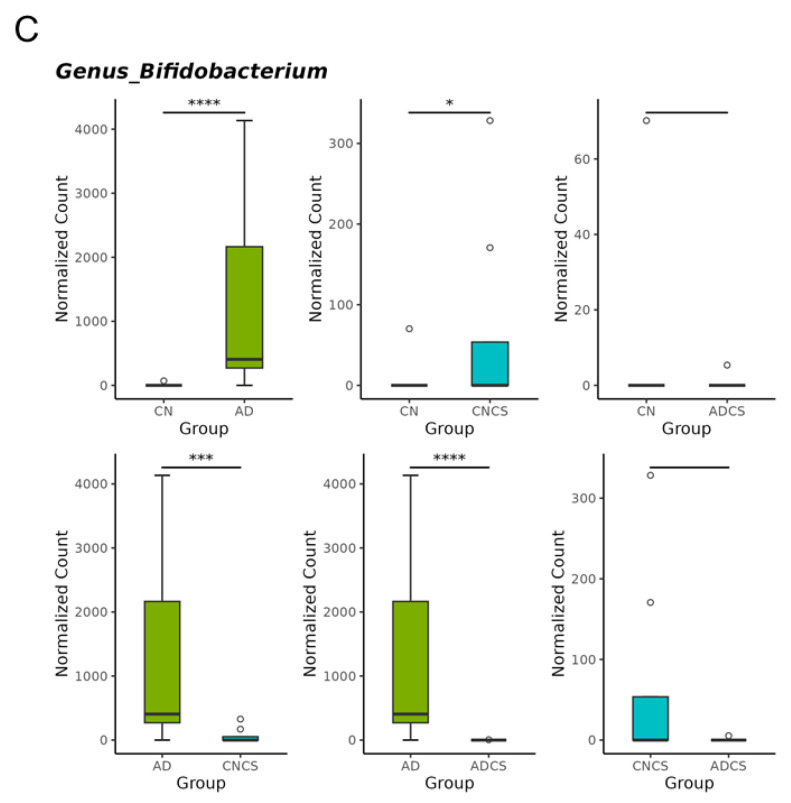
Genus-level taxa that significantly differed (false discovery rate (FDR) < 0.05) in relative abundance of (**A**) *Hungatella,* (**B**) *Anaerotruncus,* and (**C**) *Bifidobacterium*. * *p* < 0.05. *** *p* < 0.005. **** *p* < 0.001. Outliers are denoted by dots. CN = standard diet; AD = standard diet containing 0.5% adenine; CNCS = standard diet containing 3% chondroitin sulfate; ADCS = standard diet containing 0.5% adenine plus 3% CS.

Maternal adenine diet caused a decrease in genera of *Hungatella* and *Anaerotruncus* and increases in genus *Bifidobacterium* vs. the CN group (Figure 5A–C). Conversely, maternal adenine diet-induced changes were restored by CS treatment (Figure 5A–C). Compared with the CN group, maternal CS administration increased the abundance of genus *Bifidobacterium* in the CNCS group.

**Figure 6:**
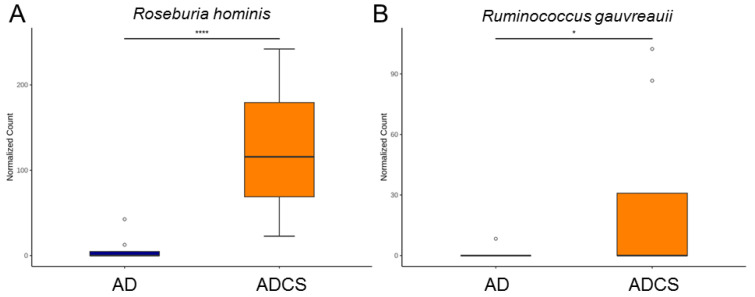
Species-level taxa that significantly differed (false discovery rate (FDR) < 0.05) in relative abundance of (**A**) *Roseburia hominis* and (**B**) *Ruminococcus gauvreauii*. * *p* < 0.05. **** *p* < 0.001. Outliers are denoted by dots. AD = standard diet containing 0.5% adenine; ADCS = standard diet containing 0.5% adenine plus 3% CS.

To further explore the beneficial effects of maternal CS treatment and delve deeper into the gut microbiota, we next highlight significant species-level changes between the AD and ADCS groups. Compared with the AD group, *Roseburia hominis* and *Ruminococcus gauvreauii* were significantly augmented in the ADCS group (Figure 6).

### 3.5. SCFAs

As shown in Table 3, maternal adenine diet led to decreased levels of acetic acid (*P*_AD_ = 0.001), butyric acid, isobutyric acid (*P*_AD_ = 0.01), butyric acid (*P*_AD_ = 0.001), isovaleric acid (*P*_AD_ < 0.001), and valeric acid (*P*_AD_ < 0.001). CS treatment has no effect on most SCFA levels, except for acetic acid, which showed a significant association with the adenine diet and CS treatment (*P*_AD×CS_ = 0.01).

## 4. Discussion

Our study offers new insights into the protective mechanisms of CS against maternal adenine diet-primed hypertension in adult male offspring, with a particular emphasis on the H_2_S pathway, gut microbiota, and NO. Our key findings reveal that (i) maternal CS supplementation averted hypertension in adult offspring that was programmed by maternal exposure to an adenine diet; (ii) CS treatment was associated with a high renal mRNA and protein abundance of 3MST; (iii) maternal CS treatment significantly increased microbiota alpha diversity and altered beta diversity in adult offspring; (iv) the maternal adenine diet caused a decrease in the genera *Hungatella* and *Anaerotruncus* and an increase in the genus *Bifidobacterium*, which was prevented by CS treatment; and (v) the advantageous action of CS against offspring hypertension correlated with increased beneficial microbes such as *Roseburia hominis* and *Ruminococcus gauvreauii*.

Supporting previous research indicating that maternal CKD leads to adverse outcomes in offspring [1,2], we observed that adult rat offspring born from dams fed an adenine diet developed hypertension and kidney dysfunction. A novel finding of this study is that supplementing the maternal diet with CS improved offspring hypertension. While several beneficial effects of CS have been reported [9], our study is the first to demonstrate that CS treatment during pregnancy and lactation can prevent hypertension in adult offspring induced by maternal adenine diet. However, CS treatment had no effect on the kidney weight-to-body weight ratio and plasma creatinine levels.

Considering that one beneficial action of CS may be attributed to the generation of H_2_S [27], our observations align with prior work supporting the effectiveness of early-life H_2_S-based interventions in averting hypertension with developmental origins [17]. CS is poorly absorbed in the intestine, unlike other sulfate molecules that are rapidly absorbed in the gut, making it more likely to be utilized by sulfate-reducing bacteria (SRB) for H_2_S production [12]. The advantageous action of CS against maternal adenine diet-induced offspring hypertension might be attributed to increased renal 3MST. Since CS administration was discontinued after weaning, its effects are owing to reprogramming rather than direct action. Although CS can directly increase gut microbial-derived H_2_S [14,15], our results extend beyond prior research by demonstrating that supplementing the maternal diet with CS may have enduring effects on the offspring’s H_2_S-generating system, enhancing H_2_S availability later in life.

The antihypertensive effect of H_2_S could be achieved via an augmentation of NO signaling [28]. This concept is reinforced by our previous study, which demonstrated that maternal sodium thiosulfate (an H_2_S donor) treatment prevented offspring hypertension by decreasing ADMA levels and increasing AAR, an index of NO availability, in a maternal adenine-diet model [8]. In the present study, a maternal adenine diet reduced AAR, which was partially restored by maternal CS treatment. Given that CS is proposed to exert anti-inflammatory and antioxidant actions by regulating NO [18], and considering the deficient NO pathway’s involvement in hypertension with developmental origins [29], the interplay between CS and NO in controlling offspring BP warrants further investigation.

Another protective effect of CS could be its influence on gut microbiota composition. A comprehensive review of human and animal studies concluded that CS supplementation did not alter gut microbial diversity but did affect the abundance of specific bacterial taxa [13]. CS exposure was associated with an increased abundance of the genus *Bacteroides* and *Desulfovibrio piger* species and a decrease in *Lactobacillus* [13]. However, our data conflict with prior studies, showing that maternal CS supplementation significantly increased microbiota alpha diversity and altered beta diversity in adult offspring, while having negligible effects on the previously mentioned taxa. These differences might be due to the discontinuation of CS supplementation after weaning, indicating that the reprogramming effects on offspring’s gut microbiota were fundamentally different from the direct effects.

Supplementing the maternal diet with adenine induced offspring hypertension, coinciding with a decrease in the genera *Hungatella* and *Anaerotruncus*. Conversely, the decrease in both taxa were restored by maternal CS treatment. Hence, the BP-lowering effects of maternal CS treatment might be related to its regulation of BP-associated taxa, as the genera *Hungatella* and *Anaerotruncus* are both reported to be negatively associated with BP [30,31].

Growing evidence suggests that probiotics have significant potential in regulating gut microbiota balance and preventing hypertension [32,33,34]. However, there is still debate regarding which probiotics and to what extent they exert effective antihypertensive benefits [35]. Supporting the prebiotic role of CS [13], our data indicated that maternal CS treatment protected against offspring hypertension by augmenting the abundance of *Roseburia hominis* and *Ruminococcus gauvreauii*, both known as next-generation probiotics [36,37]. Surprisingly, *Bifidobacterium*, a well-known probiotic, was reduced by maternal CS treatment. Previous research revealed that CS can be fermented by *Bifidobacterium* to generate SCFAs [38]. Additionally, human studies have shown that the genus *Bifidobacterium* is present at lower levels in hypertensive cases compared to control subjects [31]. Whether these differences are related to variations between humans and animal models or reprogramming versus direct effects deserves further research to fully explore CS’s functions on individual beneficial microbes and their interplay in the regulation of BP.

We further analyzed plasma SCFAs as they are involved in BP regulation [22]. Although maternal CS treatment increased acetic levels in the control group, the decreases in other SCFAs induced by maternal adenine diet was not prevented by CS. Hence, it is not clear whether the beneficial action of CS is correlated to gut microbes-derived SCFAs. Moreover, we determined microbial taxa participate in H_2_S production and metabolism. We found that CS has a minimal effect on the proportion of microorganisms with sulfite reductase, including *Bacillus*, *Corynebacterium*, *Rhodococcus*, *Klebsiella*, *Salmonella*, etc. [39]. Our data also showed that all SRBs (e.g., *Desulfobacter* or *Desulfovibrio*) were not affected by maternal CS treatment. Hence, it is unclear whether the defending role of CS is associated with changes in sulfite- or sulfate-reducing microorganisms.

We acknowledge some limitations. Firstly, the present study did not investigate how maternal CS supplementation affected the gut microbiota in dams or neonatal progeny. Understanding whether CS treatment during gestation and lactation can influence gut microbiota-derived fecal H_2_S and SCFAs, which may contribute to BP regulation in offspring later in life, requires further investigation. Another limitation is that our study only examined male offspring. It remains unknown whether there are sex differences in the effects of CS treatment, warranting additional research attention. Lastly, while our findings demonstrate the beneficial effects of CS on offspring hypertension induced by maternal adenine diet in this specific model, these results may not directly translate to other models of CKD or human populations. Therefore, additional research is necessary in diverse animal models of CKD and ultimately in human subjects before CS can be considered for clinical application.

## 5. Conclusions

In conclusion, our findings suggest that supplementing the maternal diet with CS improved offspring hypertension induced by maternal adenine diet. This improvement is likely achieved through enhanced H_2_S pathway activity and changes in gut microbiota composition. Therefore, early supplementation with chondroitin sulfate or other sulfate prebiotics may hold promise for preventing hypertension in offspring born to mothers with CKD. Considering chondroitin sulfate has been widely used for medical and nutraceutical purposes, further studies are certainly required to test its use in other models of CKD or human populations, especially in early life, to translate into clinical practice and reduce the global burden of hypertension-related diseases.

## Figures and Tables

**Table 1:** Weights, BP, and plasma creatinine level.

Groups	CN	AD	CNCS	ADCS		*p* Value	
					AD	CS	AD × CS
Body weight (BW) (g)	415 ± 18	415 ± 14	351 ± 7 *#	426 ± 9	0.007	0.046	0.007
Left kidney weight (LKW) (g)	1.83 ± 0.08	1.94 ± 0.07	1.71 ± 0.06 #	1.97 ± 0.08	0.014	NS	NS
Combined kidney weight (CKW) (g)	3.68 ± 0.16	3.94 ± 0.13	3.41 ± 0.1 #	4.02 ± 0.13	0.003	NS	NS
LKW-to-BW ratio (g/kg)	0.44 ± 0.01	0.47 ± 0.01	0.49 ± 0.01	0.47 ± 0.03	NS	NS	NS
CKW-to-BW ratio (g/kg)	0.89 ± 0.01	0.95 ± 0.02	0.97 ± 0.02	0.95 ± 0.05	NS	NS	NS
Systolic BP (mmHg)	133 ± 1	149 ± 2 *	135 ± 1 #	137 ± 1 #	<0.001	<0.001	<0.001
Diastolic BP (mmHg)	88 ± 1	91 ± 3	88 ± 2	89 ± 2	NS	NS	NS
Creatinine (μM)	16.5 ± 0.5	18.8 ± 0.8	16.2 ± 0.8	18.8 ± 0.7	0.002	NS	NS

N = 8/group; BP = blood pressure. CN = standard diet; AD = standard diet containing 0.5% adenine; CNCS = standard diet containing 3% chondroitin sulfate; ADCS = standard diet containing 0.5% adenine plus 3% CS; AD × CS = interaction of AD × CS; NS = not significant. * *p* < 0.05 vs. CN; # *p* < 0.05 vs. AD.

**Table 2:** Plasma concentrations of NO-related parameters.

Groups	CN	AD	CNCS	ADCS		*p* Value	
					AD	CS	AD × CS
L-citrulline (μM)	54.3 ± 3.3	43.0 ± 2.5	48.0 ± 1.7	46.1 ± 3.2	0.024	NS	NS
L-arginine (μM)	392.5 ± 21.6	286.2 ± 11.8	365.9 ± 18.2	302.1 ± 16.3	<0.001	NS	NS
ADMA (μM)	2.06 ± 0.1	2.19 ± 0.1	1.88 ± 0.06	2.05 ± 0.17	NS	NS	NS
SDMA (μM)	2.07 ± 0.16	2.49 ± 0.20	2.28 ± 0.10	2.02 ± 0.08	NS	NS	0.035
AAR (μM/μM)	192.9 ± 12.2	132.5 ± 7.9	197.1 ± 13.0	152.7 ± 11.8	<0.001	0.044	NS

N = 8/group; AAR = L-arginine-to-ADMA ratio; CN = standard diet; AD = standard diet containing 0.5% adenine; CNCS = standard diet containing 3% chondroitin sulfate; ADCS = standard diet containing 0.5% adenine plus 3% CS; AD × CS = interaction of AD × CS; NS = not significant.

**Table 3:** Plasma concentrations of SCFAs.

Groups	CN	AD	CNCS	ADCS		*p* Value	
					AD	CS	AD × CS
Acetic acid (μM)	943.9 ± 80.4	893.5 ± 42.3	1151 ± 49.4 *#	785.8 ± 43.8 $	0.001	NS	0.01
Propionic acid (μM)	4.04 ± 0.78	3.8 ± 0.62	5.06 ± 0.27	4.59 ± 0.56	NS	NS	NS
Isobutyric acid (μM)	2.12 ± 0.12	1.75 ± 0.14	2.31 ± 0.09	1.9 ± 0.19	0.01	NS	NS
Butyric acid (μM)	10.98 ± 0.49	9.29 ± 0.4	10.45 ± 0.53	8.73 ± 0.31	0.001	NS	NS
Isovaleric acid (μM)	14.42 ± 2	7.62 ± 0.47	11.99 ± 0.67	6.67 ± 0.5	<0.001	NS	NS
Valeric acid (μM)	3.88 ± 0.12	3.17 ± 0.19	4 ± 0.13	3.36 ± 0.14	<0.001	NS	NS

N = 8/group; CN = standard diet; AD = standard diet containing 0.5% adenine; CNCS = standard diet containing 3% chondroitin sulfate; ADCS = standard diet containing 0.5% adenine plus 3% CS; AD × CS = interaction of AD × CS; NS = not significant. * *p* < 0.05 vs. CN; # *p* < 0.05 vs. AD; $ *p* < 0.05 vs. CNCS.

## Data Availability

The data that support the findings of this study are contained within the article.

## References

[1] Munkhaugen J., Lydersen S., Romundstad P.R., Widerøe T.-E., Vikse B.E., Hallan S. (2009). Kidney function and future risk for adverse pregnancy outcomes: A population-based study from HUNT II, Norway. Nephrol. Dial. Transplant..

[2] Piccoli G.B., Alrukhaimi M., Liu Z.H., Zakharova E., Levin A., World Kidney Day Steering Committee (2018). What we do and do not know about women and kidney diseases; Questions unanswered and answers unquestioned: Reflection on World Kidney Day and International Woman’s Day. Physiol. Int..

[3] Fleming T.P., Velazquez M.A., Eckert J.J. (2015). Embryos, DOHaD and David Barker. J. Dev. Orig. Health Dis..

[4] Hanson M.A., Gluckman P.D. (2014). Early developmental conditioning of later health and disease: Physiology or pathophysiology?. Physiol. Rev..

[5] Paauw N.D., van Rijn B.B., Lely A.T., Joles J.A. (2017). Pregnancy as a critical window for blood pressure regulation in mother and child: Programming and reprogramming. Acta Physiol..

[6] Diwan V., Brown L., Gobe G.C. (2018). Adenine-induced chronic kidney disease in rats. Nephrology.

[7] Hsu C.N., Yang H.W., Hou C.Y., Chang-Chien G.P., Lin S., Tain Y.L. (2020). Maternal Adenine-Induced Chronic Kidney Disease Programs Hypertension in Adult Male Rat Offspring: Implications of Nitric Oxide and Gut Microbiome Derived Metabolites. Int. J. Mol. Sci..

[8] Tain Y.L., Hou C.Y., Chang-Chien G.P., Lin S., Hsu C.N. (2023). Protection by Means of Perinatal Oral Sodium Thiosulfate Administration against Offspring Hypertension in a Rat Model of Maternal Chronic Kidney Disease. Antioxidants.

[9] Shen Q., Guo Y., Wang K., Zhang C., Ma Y. (2023). A Review of Chondroitin Sulfate’s Preparation, Properties, Functions, and Applications. Molecules.

[10] Meng Z., Liu J., Zhou N. (2023). Efficacy and safety of the combination of glucosamine and chondroitin for knee osteoarthritis: A systematic review and meta-analysis. Arch. Orthop. Trauma Surg..

[11] Stellavato A., Restaino O.F., Vassallo V., Finamore R., Ruosi C., Cassese E., De Rosa M., Schiraldi C. (2019). Comparative Analyses of Pharmaceuticals or Food Supplements Containing Chondroitin Sulfate: Are Their Bioactivities Equivalent?. Adv. Ther..

[12] Barthe L., Woodley J., Lavit M., Przybylski C., Philibert C., Houin G. (2004). In vitro intestinal degradation and absorption of chondroitin sulfate, a glycosaminoglycan drug. Arzneimittel-Forschung.

[13] Shmagel A., Demmer R., Knights D., Butler M., Langsetmo L., Lane N.E., Ensrud K. (2019). The Effects of Glucosamine and Chondroitin Sulfate on Gut Microbial Composition: A Systematic Review of Evidence from Animal and Human Studies. Nutrients.

[14] Pichette J., Fynn-Sackey N., Gagnon J. (2017). Hydrogen Sulfide and Sulfate Prebiotic Stimulates the Secretion of GLP-1 and Improves Glycemia in Male Mice. Endocrinology.

[15] Chen L., Gao Y., Zhao Y., Yang G., Wang C., Zhao Z., Li S. (2022). Chondroitin sulfate stimulates the secretion of H_2_S by Desulfovibrio to improve insulin sensitivity in NAFLD mice. Int. J. Biol. Macromol..

[16] Kimura H. (2015). Signaling molecules: Hydrogen sulfide and polysulfide. Antioxid. Redox Signal..

[17] Hsu C.N., Tain Y.L. (2021). Preventing Developmental Origins of Cardiovascular Disease: Hydrogen Sulfide as a Potential Target?. Antioxidants.

[18] Vallières M., du Souich P. (2010). Modulation of inflammation by chondroitin sulfate. Osteoarthr. Cartil..

[19] Reckelhoff J.F. (2001). Gender differences in the regulation of blood pressure. Hypertension.

[20] Schmittgen T.D., Livak K.J. (2008). Analyzing real-time PCR data by the comparative C(T) method. Nat. Protoc..

[21] Bode-Böger S.M., Scalera F., Ignarro L.J. (2007). The L-arginine paradox: Importance of the L-arginine/asymmetrical dimethylarginine ratio. Pharmacol. Ther..

[22] Pluznick J.L. (2017). Microbial short-chain fatty acids and blood pressure regulation. Curr. Hypertens. Rep..

[23] Regueira-Iglesias A., Balsa-Castro C., Blanco-Pintos T., Tomás I. (2023). Critical review of 16S rRNA gene sequencing workflow in microbiome studies: From primer selection to advanced data analysis. Mol. Oral Microbiol..

[24] Bolyen E., Rideout J.R., Dillon M.R., Bokulich N.A., Abnet C.C., Al-Ghalith G.A., Alexander H., Alm E.J., Arumugam M., Asnicar F. (2019). Reproducible, interactive, scalable and extensible microbiome data science using QIIME 2. Nat. Biotechnol..

[25] Price M.N., Dehal P.S., Arkin A.P. (2010). FastTree 2—Approximately maximum-likelihood trees for large alignments. PLoS ONE.

[26] Segata N., Izard J., Waldron L., Gevers D., Miropolsky L., Garrett W.S., Huttenhower C. (2011). Metagenomic biomarker discovery and explanation. Genome Biol..

[27] Rey F.E., Gonzalez M.D., Cheng J., Wu M., Ahern P.P., Gordon J.I. (2013). Metabolic niche of a prominent sulfate-reducing human gut bacterium. Proc. Natl. Acad. Sci. USA.

[28] Wang R. (2023). Roles of Hydrogen Sulfide in Hypertension Development and Its Complications: What, So What, Now What. Hypertension.

[29] Tain Y.L., Hsu C.N. (2016). Targeting on Asymmetric Dimethylarginine-Related Nitric Oxide-Reactive Oxygen Species Imbalance to Reprogram the Development of Hypertension. Int. J. Mol. Sci..

[30] Jin L., Cui X., Cai J. (2018). Study of gut microbiota in over-weighted and obese hypertensive patients. Infect. Dis. Informat..

[31] Guo Y., Li X., Wang Z., Yu B. (2021). Gut Microbiota Dysbiosis in Human Hypertension: A Systematic Review of Observational Studies. Front. Cardiovasc. Med..

[32] Khalesi S., Sun J., Buys N., Jayasinghe R. (2014). Effect of probiotics on blood pressure: A systematic review and meta-analysis of randomized, controlled trials. Hypertension.

[33] Chen Z., Liang W., Liang J., Dou J., Guo F., Zhang D., Xu Z., Wang T. (2023). Probiotics: Functional food ingredients with the potential to reduce hypertension. Front. Cell Infect. Microbiol..

[34] Hsu C.N., Lin Y.J., Hou C.Y., Tain Y.L. (2018). Maternal Administration of Probiotic or Prebiotic Prevents Male Adult Rat Offspring against Developmental Programming of Hypertension Induced by High Fructose Consumption in Pregnancy and Lactation. Nutrients.

[35] Yuan L., Li Y., Chen M., Xue L., Wang J., Ding Y., Gu Q., Zhang J., Yang R., Zhao H. (2023). Effects of probiotics on hypertension. Appl. Microbiol. Biotechnol..

[36] Cheng H.L., Yen G.C., Huang S.C., Chen S.C., Hsu C.L. (2022). The next generation beneficial actions of novel probiotics as potential therapeutic targets and prediction tool for metabolic diseases. J. Food Drug Anal..

[37] Kumari M., Singh P., Nataraj B.H., Kokkiligadda A., Naithani H., Azmal Ali S., Behare P.V., Nagpal R. (2021). Fostering next-generation probiotics in human gut by targeted dietary modulation: An emerging perspective. Food Res. Int..

[38] Wei C.Y., Liao N.B., Zhang Y., Ye X.Q., Li S., Hu Y.Q., Liu D.H., Linhardt R.J., Wang X., Chen S.G. (2017). In vitro fermentation behaviors of fucosylated chondroitin sulfate from Pearsonothuria graeffei by human gut microflora. Int. J. Biol. Macromol..

[39] Tomasova L., Konopelski P., Ufnal M. (2016). Gut Bacteria and Hydrogen Sulfide: The New Old Players in Circulatory System Homeostasis. Molecules.

